# Polycystic Ovary Syndrome and Irritable Bowel Syndrome: Is There a Common Pathway?

**DOI:** 10.1002/edm2.477

**Published:** 2024-03-17

**Authors:** Marzieh Saei Ghare Naz, Vida Ghasemi, Shabahang Amirshekari, Fahimeh Ramezani Tehrani

**Affiliations:** ^1^ Reproductive Endocrinology Research Center, Research Institute for Endocrine Sciences Shahid Beheshti University of Medical Sciences Tehran Iran; ^2^ Asadabad School of Medical Sciences Asadabad Iran; ^3^ The Foundation for Research & Education Excellence Vestavia Hills Alabama USA

**Keywords:** irritable bowel syndrome, metabolic syndrome, polycystic ovary syndrome, review

## Abstract

**Objective:**

Little is known about how polycystic ovary syndrome (PCOS) is linked to irritable bowel syndrome (IBS). This study aimed to review the existing literature regarding the association between PCOS or its symptoms and complications with IBS.

**Methods:**

In this review, studies that investigated the proposed cross‐link between features of PCOS and IBS were included. This review collectively focused on recent findings on the mechanism and novel insight regarding the association between IBS and PCOS in future clinical practice. An electronic search of PubMed, Scopus, Epistemonikos, Cochrane Library and Google Scholar was performed. We did not restrict the study setting and publication date.

**Results:**

The existing evidence has not completely answered the question of whether there is an association between PCOS and IBS and vice versa. Six case–control studies (793 women with PCOS and 547 women in the control group) directly assessed the association between PCOS and IBS. The prevalence of IBS among women with PCOS in these studies has ranged from 10% to 52% compared with 5%–50% in control groups. Evidence suggested the common pathways may have contributed to the interaction between IBS and PCOS, including metabolic syndrome, sex hormone fluctuation, dysregulation of neurotransmitters, psychological problems and environmental and lifestyle factors. To date, it is still ambiguous which of the mentioned components largely contributes to the pathogenesis of both.

**Conclusion:**

Although limited evidence has shown a higher prevalence of IBS in women with PCOS, there are several potential, direct and common indirect pathways contributing to the development of both IBS and PCOS.

## Graphical abstract

Although limited evidence has shown a higher incidence of IBS in women with PCOS, there are several potential direct and common indirect pathways contributing to the development of both IBS and PCOS.

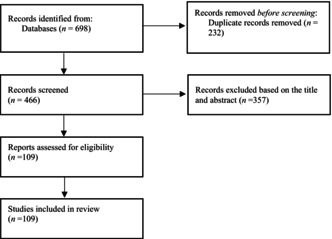

## 1 Introduction

Polycystic ovary syndrome (PCOS) is the most common reproductive disorder around the world. PCOS is mainly characterised by hyperandrogenism and anovulation [1, 2]. This endocrine disorder is linked to some short‐ and long‐term complications, including cardio‐metabolic, obstetric, oncology and psychological complications [3, 4]. It has been proposed that gastrointestinal dysbiosis can play a role in the pathophysiology of PCOS [5]. PCOS patients are more prone to experience gastrointestinal (GI) disturbances such as irritable bowel syndrome (IBS) [6, 7]. A recent meta‐analysis showed that the risk of IBS in women with PCOS is two times higher in the than control group [8].

IBS is one of the most prevalent functional bowel disorders, and its prevalence varies widely in different countries according to diagnostic criteria [9]. The Rome IV diagnostic criteria defined IBS as recurrent abdominal pain that is associated with a change in bowel habits or defecation. Disordered bowel habits are typically present (i.e. constipation, diarrhoea or a mix of constipation and diarrhoea), as are symptoms of abdominal bloating/distension. The symptoms started 6 months prior to the diagnosis, and they should have persisted for 3 months at this point [10]. Gender differences in IBS symptom severity are influenced by female sex hormones [11]. Indeed, the impact of the fluctuation of sex hormones which appears in relation to pregnancy, menstrual cycle or menopausal states on gastrointestinal disorders was addressed previously [11, 12].

Genetic factors, in combination with epigenetic, environmental and peripheral factors, contribute to the development of IBS [13]. The increased prevalence of IBS among females with PCOS [6, 14] could be explained in several ways including the fluctuation of sex hormones. In the light of this fact, IBS is highly correlated with metabolic syndrome (METs) [15]. Similarly, women with PCOS are at high risk of developing METs [16]. Moreover, it has been reliably demonstrated that there is a link between psychological morbidity and symptoms of both IBS and PCOS [17].

Due to the lack of evidence, it is unclear whether it is PCOS in women that make them susceptible to the increased risk of developing IBS or vice versa. Hence, this review aimed to summarise the key evidence that supports the putative association between PCOS or its symptoms and complications with IBS. It is hoped that this study could shed light on the direction of future studies.

## 2 Methods

This narrative review aimed to determine the association between PCOS and IBS and the common pathway between them. We searched PubMed, Scopus, Epistemonikos, Cochrane Library: Cochrane Reviews and Google Scholar for all kinds of studies showing the link between PCOS and IBS till December 2023. In this narrative review, human and animal studies (clinical trials, review and observational studies) investigating the association between clinical (anthropometric, reproductive and metabolic factors) and biochemical characteristic features of PCOS and IBS were included. Also, we excluded case reports, commentaries, editorials and letters to the editor. We did not restrict the study setting and publication date; however, there was a restriction in this study regarding the English language. The search was performed around the key terms, including polycystic ovary syndrome, PCOS, Stein‐Leventhal Syndrome, Sclerocystic Ovarian Degeneration, Sclerocystic Ovary Syndrome, Ovarian Syndrome Polycystic, Irritable Bowel Syndromes, Colon Irritable, Irritable Colon, Colitis, Mucous Colitis, Gastrointestinal Diseases, Colonic Diseases, Colonic Diseases, Functional, Intestinal Diseases.

We identified additional studies through a manual search of the bibliographic references of relevant articles and existing reviews. Articles that met the inclusion criteria were carefully read and, when appropriate, further articles retrieved from their references were also reviewed with the aim to include other critical studies that might have been missed in the initial search. Qualitative studies, which directly assessed the association between PCOS and IBS, were performed utilising the Newcastle–Ottawa Scale for case–control studies. This scale was used to assess the selection, comparability and exposure domains [18]. In this study, scores <3, between 3 and 5 and >6 were considered as low, moderate and high‐quality studies.

## 3 Overview of the Relationship Between PCOS and IBS

**FIGURE 1:**
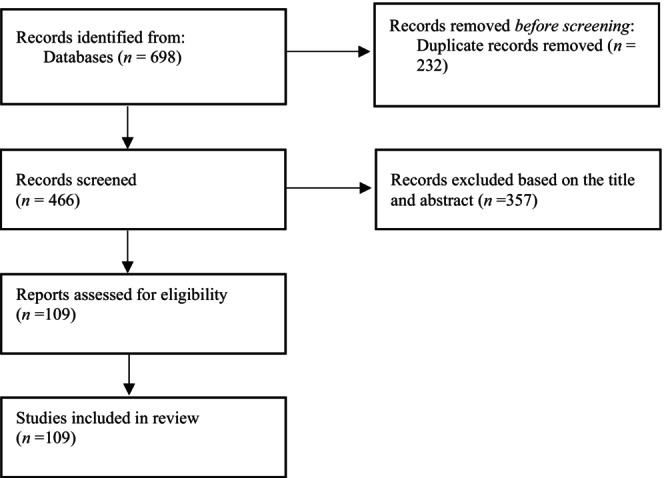
Flow chart of included studies.

Figure 1 shows a flow chart of the included studies. So far, according to our knowledge, there are six case–control published articles that have directly investigated the association between PCOS and IBS. Overall, these studies included 793 women with PCOS and 547 women in the control group (Table 1). All of these studies had moderate to high‐quality score. In two studies, the prevalence of IBS was similar in women with PCOS compared with the controls [6, 7]. The results of Kałużna et al.'s study showed that the prevalence of IBS symptoms in patients with PCOS was not different from that in the control group. In addition, hyperandrogenism and obesity in patients with PCOS had no effect on the occurrence of IBS symptoms, and hormonal, anthropometric and chemical differences were not seen between IBS‐PCOS and non‐IBS‐PCOS patients. But in this study, the prevalence of metabolic syndrome and depression was higher in IBS‐PCOS than in non‐IBS‐PCOS patients [6].

**TABLE 1:** Characteristics of included studies related the PCOS and IBS.

Author, year, reference	Study design	Participants	PCOS diagnosis criteria	IBS diagnosis criteria	Findings	Quality score			
						Selection	Comparability	Exposure	Total
Dursun et al. (2018) [19]	Case–control	Patients with PCOS (*n* = 54) Controls (*n* = 53)	Revised 2003 Rotterdam criteria	Rome III criteria	Prevalence of IBS was 39% in PCOS patients vs 19% in control	2	1	2	5
Mathur et al. (2010) [20]	Case–control	Patients with PCOS (*n* = 36) Controls (*n* = 29)	NIH 1990	Bowel‐related questions	Prevalence of IBS was 41.7% in PCOS patients vs 10.3% in control	4	—	2	6
Kałużna et al. (2022) [6]	Case–control	Patients with PCOS (*n* = 133) Controls (*n* = 72)	ESHRE guideline	Rome IV criteria	Prevalence of IBS was 24% (32/133) in PCOS patients vs. 21% in control (15/72) (*p* = 0.60)	3	2	3	8
Bazarganipour et al. (2020) [14]	Case–control	Patients with PCOS (*n* = 101) Controls (*n* = 100)	Rotterdam diagnostic criteria	Rome III criteria	IBS symptoms were higher in PCOS (20.7%) than control group (11%) (*p* = 0.05).	3	—	3	6
Tseng et al. (2020) [17]	Case–control	Patients with PCOS (*n* = 431) Controls (*n* = 259)	Rotterdam diagnostic criteria	Rome III criteria	Women with PCOS were more likely to have IBS (10.7% vs. 5.8%, *p* = 0.029) and obesity (29% vs. 4%, *p* < 0.001) than healthy volunteers. Mixed‐type IBS (IBS‐M) was the most common subtype (74%) among patients with PCOS and IBS.	3	—	3	6
Naziye Gürkan et al. (2022) [7]	Case–control	Women with PCOS (*n* = 38) and control group (*n* = 34)	Rotterdam Criteria	Roma IV	IBS prevalence was similar in PCOS (52%) and the control group (50%).	3	—	2	5

Abbreviations: ESHRE, European Society of Human Reproduction and Embryology; IBS, irritable bowel syndrome; NIH, National Institutes of Health; PCOS, polycystic ovary syndrome.

In the study by Gürkan, Mehmet, and Gürbüz [7] the prevalence of IBS was similar in PCOS and control groups. In addition in the IBS‐PCOS group, fasting insulin (FI) and luteinizing hormone (LH) were significantly lower than in the non‐IBS‐PCOS group (*p* < 0.05), but there was no statistically significant association between IBS‐PCOS and non‐IBS‐PCOS in terms of gastrointestinal symptoms.

In the study by Tseng et al. [17] women with PCOS were more likely to have IBS. In addition, in women with PCOS and IBS, sleep difficulties and psychiatric morbidities were more prevalent compared to PCOS patients without IBS, but anthropometric, metabolic and hormonal profiles were similar between IBS‐PCOS and non‐IBS‐PCOS patients.

Bazarganipour et al. [14] in their study, in addition to showing that the prevalence of IBS is higher in patients with PCOS than that in the control group, stated that the quality of life in patients who had both IBS and PCOS at the same time was lower than in other groups.

## 4 Potential Pathways That Link PCOS and IBS

**FIGURE 2:**
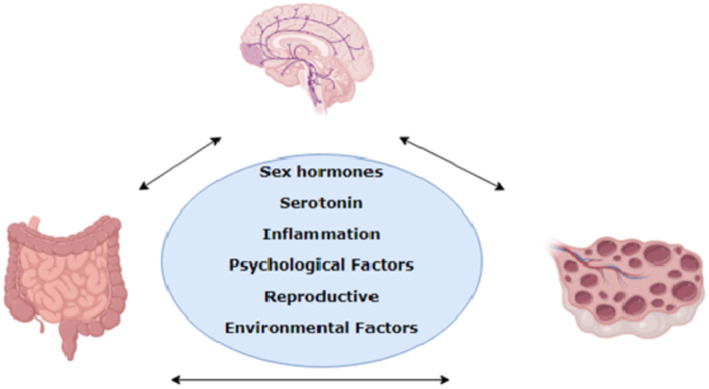
Cross‐link between IBS and PCOS.

Figure 2 depicts the potential cross‐link between IBS and PCOS.

### 4.1 Sex Hormone Alterations

The link between sex hormones and IBS has gained growing interest over the last decade. Evidence emphasises that women are more susceptible to IBS than men [21]. Houghton et al. [22] reported that testosterone levels are inversely associated with IBS symptomatology. Although the main role of sex hormones is in human reproduction, their role in other organs has been demonstrated. The role of sex hormones in GI tract motility function has been evidenced in the recent literature [23, 24, 25]. This observation suggests that if hormonal changes occur throughout the female's life (pregnancy, menopause, etc.), these alterations could affect the GI function [23]. Generally, hormonal changes during the hormonal transition phase of menopause might be relevant to gastrointestinal health and IBS. Interestingly, Ruigomez et al. [26] in their study observed that postmenopausal women who are under hormone therapy are at increased risk of IBS similar to premenopausal women. It is also well known that the fluctuation of sex hormones during the menstrual cycle could affect the severity of symptoms of IBS [24], which highlights the important role of sex hormones in the activation of the pathological pathways of IBS.

Kim et al. [27] in their study among young males demonstrated that testosterone and sex hormone‐binding globulin (SHBG) levels in patients with IBS were higher than in the control group, which reflects the differing status of sex hormones in patients with IBS. It is surprising that sex hormones can affect not only the susceptibility to IBS, but also pain perception. Evidence has indicated that oestrogen receptors (OR) and androgen receptors (AR) commonly act as central nervous system (CNS) stimulants and inhibition, respectively, hence variations in sex hormone levels are able to alter the symptoms of IBS [21, 28]. Previously, the results of a meta‐analysis demonstrated that the symptoms of IBS among women with and without IBS were commonly reported in menses rather than in other phases of the menstrual cycle [11]. Furthermore, other reviews concluded that oestrogen and progesterone withdrawal around menopause and menses might contribute to increased symptoms of GI [29]. The lower levels of progesterone during menses, is thought to contribute to greater somatic pain [29].

Gynaecological complaints are more commonly reported by women with IBS [30]. A population‐based study revealed that women with dysmenorrhoea are more prone to IBS symptoms [31]. Oestrogen plays an important role in visceral afferent, autonomic nervous system and pain pathways [32]. Far less research has investigated the fluctuation of hormones and IBS in women with PCOS. Apart from this, ovarian hormones could also contribute to physiological responses and coping behaviour against stress [33]. It is recommended that in women with PCOS, more attention should be paid to the history of menstrual cycle irregularity [34]. To sum up, these findings indicated that pathophysiological changes in sex hormone levels may play a role in the development of IBS in women with PCOS.

### 4.2 Serotonin Dysfunction

Neurotransmitter alteration could be considered another factor suggested to be contributing to the development of both PCOS and IBS. It has been reported that serotonin dysregulation is involved in the pathophysiology of PCOS [35]. Similarly, evidence has been shown that the release of serotonin is disturbed in patients with IBS [36]. The evidence revealed that the decrease in intestinal serotonin leads to weakness in the intestinal lining, which inevitably results in constipation and an increase in serotonin levels within the gut [37]. Another hypothesis in this context implies the deficiency of the serotonin transporter‐serotonin reuptake transporter (SERT) enterocytes in IBS patients [38]. Indeed, serotonin, which is regulated by SERT, could influence gut distension, motility and visceral sensitivity [39]. So, any disturbance in serotonin levels could alter the development of IBS. However, animal studies demonstrated that gut dysbiosis can result in insulin resistance in the PCOS mouse model [40].

What's more, oral contraceptive (OCs) are the first‐line treatment option in the management of PCOS. A recent study has shown that in women who use OCs, global brain serotonin four receptor binding was 9%–12% lower than in women not using this agent; it is speculated that the reduction in ovarian hormones could result in a reduction in 5‐HT4R gene expression [41]. So, this is one possible cause of increased risk of IBS in women with PCOS.

Today, researchers are exploring novel pharmacological approaches such as 5‐HT3 receptor antagonists and 5‐HT4 receptor agonists for IBS management [42]. Furthermore, scientists reported the positive effect of α‐lactalbumin maintaining high levels of serotonin in the management of PCOS [43].

### 4.3 Inflammatory Factors

Inflammation is a component that greatly contributes to the pathogenesis of both PCOS and IBS. Mucosal inflammation and neuroinflammation are more likely involved in the pathophysiology of IBS [44]. In addition, similar findings showed the role of high‐sensitive C‐reactive protein in both PCOS [45, 46] and IBS [47]. With PCOS, this inflammation marker is associated with obesity [45] and cardiovascular disease [46].

Recently, Parker, O'Brien, and Hawrelak [5] in a narrative review attempted to present the role of gastrointestinal symbiosis and revealed that lipopolysaccharide (LPS) and LPS‐binding protein (LPS‐BP) might be involved in the pathogenesis of PCOS. Dlugosz et al. [48] emphasised the important role of LPS in the development of IBS. Inflammation could alter the SERT, which in turn decreases serotonin levels and acts as a trigger for IBS [39].

Moreover, inflammation (inflammation induced by diet, adipose tissue and chronic low‐grade inflammation) and oxidative stress could result in insulin resistance and ovarian dysfunction in women with PCOS [49]. Also, in IBS, inflammation and oxidative stress, as well as stress‐modulating pathways, can be related to the development of gastrointestinal symptoms of IBS. Generally, the main disorder that definitively contributes to the pathology of IBS may be two‐way communication errors of the gut–brain axis [50].

### 4.4 Gut–Brain Alteration

The role of the gut–brain axis in the pathogenesis of IBS and d PCOS has drawn much attention recently [51, 52, 53, 54]. Gut microbiome alteration in women with PCOS might be closely linked to insulin resistance, sex hormone levels, and immune change function and inflammation [21, 55]. Furthermore, gut microbiome by mediating the systemic low‐grade inflammation and insulin resistance in women with PCOS contributes to the development of PCOS [56]. In fact, gut microbiota plays an important role in the incidence, progression and phenotype of PCOS [57, 58].

Gut microbial symbiosis may be responsible for neuroendocrine alteration in women with PCOS [53, 59]. Similarly, the gut–brain alteration was also observed in patients with IBS. Evidence suggests that gut inflammation, cytokine response, and the gut microbiome contribute to such gut‐to‐brain changes in IBS [51, 60]. Therefore, when it comes to the gut–brain axis it is hypothesised that both IBS and PCOS share a common pathway.

Recently, scientists demonstrated the positive effect of bacteriotherpeutics like probiotics, synbiotics and faecal microbiota transplant (FMT) in PCOS and IBS [61, 62, 63].

### 4.5 Metabolic Disturbances

Numerous studies have been postulated to explain the role of metabolic parameters in the pathogenesis of both PCOS and IBS. It is well documented that women with PCOS are more likely to suffer from METs [64]. The results of a review on humans supported these findings and demonstrated that insulin resistance, obesity, dyslipidaemia and hyperandrogenism contributing factors to the metabolic syndrome in PCOS [65]. Although some researchers have supported the association between IBS and METs [15, 66], others have found no association between IBS and METs [67]. Subsequent observational studies on the relationship between Mets and IBS and PCOS are needed.

There is an established link between IBS and prediabetes/diabetes [68] as well as PCOS and diabetes [69]. Furthermore, co‐incidence of nonalcoholic fatty liver disease with both IBS and PCOS [70, 71] has been supported recently. The recognition of the mechanisms behind these observations is unclear.

Obesity is a possible common comorbidity of PCOS and IBS [72, 73]. As the aforementioned evidence seems to support the role of obesity in the pathogenesis of IBS and PCOS, it should be expected that any weight reduction would have a beneficial effect on IBS and PCOS. It is also reported that inflammation, physical inactivity, microbiota, diet and psychological factors could mediate the association between obesity and IBS [74, 75].

Collectively, metabolic abnormality not only acts as a mechanical effect but also plays the role of risk factor for the close link between PCOS and IBS. Further studies are needed to deepen our understanding in this regard.

### 4.6 Psychological Stress

One of the key elements for developing PCOS and IBS is psychological stress. Stress could act as a trigger for IBS via activation of the neuro‐endocrine‐immune pathways and following it gut–brain axis and microbiota‐gut‐brain axis [13, 76]. Roohafza et al. suggested a high prevalence of mental disorders like stress, anxiety and depression in subjects with IBS [77]. In IBS, impairment in the brain–gut pathway results in psychological manifestations of the disease. A systematic review revealed that individuals with IBS highly suffer from depression and anxiety. It is said that both PCOS and IBS are stress‐sensitive disorders. The stress‐induced alteration could adversely affect the bacterial composition of the GI tract [78].

Furthermore, omen who use OCs are at risk for depressive symptoms [79, 80], which can be altered for the development of IBS. Conversely, there is also evidence which demonstrated that using OC was not associated with mood disorders [81]. Nevertheless, as adolescent females start on OCs at an increasingly young age, (12) their brains are more susceptible to the potential impact of exogenous hormones as they go through critical stages of brain maturation (13). Mood disorders like depression and anxiety were associated with an increased risk of developing IBS [82].

Evidence also reported that the elevated cortisol/dehydroepiandostrerone ratio after waking up was observed among individuals with IBS compared to the non‐IBS ones [83]. This highlights the effect of long‐term stress. Like IBS, stress is also an important component of PCOS [84]. A meta‐analysis among women with PCOS has shown higher levels of cortisol as a potential stress marker in the pathogenesis of PCOS [85]. Other animal models demonstrated that long‐term stress in rat models could induce the PCOS phenotype [86].

### 4.7 Reproductive Disturbances

It has been proposed that in women with IBS the odds of conception might be decreased due to the putative mechanism of oxidative stress [87]. Furthermore, women with IBS are more prone to fertility problems including miscarriage and ectopic pregnancy [88, 89]. The result of another study among 9,096,788 deliveries demonstrates that women with IBS have a higher risk of developing adverse pregnancy outcomes [90].

Similar to women with IBS, women with PCOS are at risk of infertility, especially ovulatory infertility. Difficulty in conceiving is a common reproductive problem of women with PCOS [91]. In addition, it is associated with an increased risk of pregnancy complications, such as abortion, gestational diabetes and preeclampsia [92, 93]. On the contrary, women with PCOS are more prone to suffer from being overweight and obese; these factors themselves cause the decreased possibility of fertility with impaired ovulation, quality of oocyte and embryo implantation. In addition, if women with PCOS use assisted reproductive technology (ART), the rates of implantation, pregnancy and live birth will decrease, whereas, the risk of miscarriage will increase [94, 95].

The prevalence of obesity and body fat percentage is higher in women with PCOS and IBS than in women with PCOS alone, [20] and obesity/overweight simultaneously increases the risk of sub‐fecundity, infertility, miscarriage, poor ART outcome and decreases live birth rate in the former [96].

The prevalence of insulin resistance, impaired glucose intolerance (IGT) and diabetes mellitus is higher in women with PCOS than in healthy women which can induce the reproductive traits of PCOS [97, 98].

### 4.8 Environmental and Lifestyle Factors

There is more evidence that shows that environmental toxins have a significant impact on reproduction as well as gastrointestinal disturbances [99, 100]. Advanced glycation end products (AGEs) are examples of environmental factors in the development or progression of PCOS and IBS [101]. Prepared fast food and cooking food at very high temperatures can increase the AGEs, which have an adverse effect on the pathophysiology of PCOS and IBS [102]. The literature showed that AGEs are associated positively with insulin resistance, testosterone and anti‐Müllerian hormone levels [102, 103]. In addition, in animals fed enriched AGE diets showed hormonal and metabolic disorders and an accumulation of AGEs in the ovarian tissue and [104]. On the contrary, the low AGE diet seemed to have a beneficial effect on oxidative stress in PCOS [105]. The literature showed that most dietary receptors of AGEs accumulate in the ileum and colon [106]. These AGEs by reducing enzymatic antioxidant pathways and increasing the level of inflammatory cytokines can reduce the first‐line antioxidant defence and stimulate the inflammatory response in the gastrointestinal tract [107].

Endocrine disrupting chemicals (EDCs) are other environmental toxins in the environment, food, personal care products and manufactured products that affect the reproductive and health system and interfere with hormones that are responsible for homeostasis, reproduction and developmental process [108]. The results of a study by Eleni Kandaraki [109] showed the levels of bisphenol A (BPA), the most common chemical produced worldwide and one of the most widely studied EDC, were significantly higher in the PCOS group than in the controls. The main role of BPA in the pathogenesis of PCOS is still not well understood, but there are many reports in regard to the effect of these EDSs on ovarian steroidogenesis [110].

Diet as a main component of lifestyle plays role in both pathophysiology as well as treatment of IBS [111] and PCOS [112], so diet therapy plays an important role in controlling and improving symptoms of IBS and PCOS [113]. Evidence has demonstrated that lifestyle modification is the cornerstone for the management of IBS and PCOS [113, 114, 115].

For example, the result of a systematic review showed that the Dietary Approaches to Stop Hypertension (DASH) is most effective in insulin resistance in PCOS [113], which is in line with the results of previous studies in patients with type 2 diabetes [116]. In IBS patients, the general recommendation is consuming a regular diet, exercising, physical activity, drinking enough water, avoiding spicy and fatty foods and following a low FODMAP diet [117].

Stress as a lifestyle factor plays an important role in the development and severity of IBS symptoms [76]. It can be due to the effect of stress on the brain–gut interactions through the change in the activity of the hypothalamic–pituitary–adrenal (HPA) axis and of the autonomic nervous (ANS), metabolic and immune systems [118]. In addition, stress stimulates the sympathetic nervous system and releases ACTH and cortisol, which affect gut function [76].

Like IBS, in the PCOS population also stress is an important component [84]. The main role of catecholamine in response to stress in the brain can be the main cause of mental and metabolic disorders in PCOS [119].

### 4.9 Limitation and Strength

The main strength of this review lies in presenting the factors that are common in the pathological pathways of PCOS and IBS. This review sets the background for future biological studies that aim to clear understanding the aetiology of the coincidence of PCOS–IBS. This review mainly is limited by observational studies that used different criteria for diagnosis and does not fully considered some confounders. What's, there is a lack of knowledge regarding the association between different phenotypes of PCOS and IBS. To better understand these, future studies with a large sample size are recommended. Furthermore, in this study we did not include grey literature.

### 4.10 Conclusion

This study aimed to shed light on the crosstalk between PCOS and IBS. The existing evidence has not completely answered the question of an association between PCOS and IBS and vice versa, and a few studies have shown a higher prevalence of IBS in women with PCOS. Despite that, several common potential pathways directly and indirectly may contribute to the interaction between IBS and PCOS, including alteration in sex hormones or gut–brain, dysregulation of neurotransmitters and inflammatory factors, metabolic or reproductive disturbances, and psychological, environmental and lifestyle factors.

## Author Contributions

**Marzieh Saei Ghare Naz:** Designed and directed the project; writing of the manuscript; methodology; review and edit the manuscript. **Vida Ghasemi:** Investigation (equal); writing – original draft (equal); writing – review and editing (equal). **Shabahang Amirshekari:** Methodology (equal); writing – original draft (equal); writing – review and editing (equal). **Fahimeh Ramezani Tehrani:** Designed and directed the project; supervised the work; writing of the manuscript; methodology; and review and edit the manuscript.

## Ethics Statement

The study was approved by the ethics committee of the Research Institute for Endocrine Sciences, Shahid Beheshti University of Medical Sciences.

## Consent

Not applicable.

## Conflicts of Interest

The authors declare no conflicts of interest.

## Data Availability

Not applicable.
